# The dietary treatment of histamine intolerance reduces the abundance of some histamine-secreting bacteria of the gut microbiota in histamine intolerant women. A pilot study

**DOI:** 10.3389/fnut.2022.1018463

**Published:** 2022-10-21

**Authors:** Sònia Sánchez-Pérez, Oriol Comas-Basté, Adriana Duelo, M. Teresa Veciana-Nogués, Mercedes Berlanga, M. Carmen Vidal-Carou, M. Luz Latorre-Moratalla

**Affiliations:** ^1^Departament de Nutrició, Ciències de l’Alimentació i Gastronomia, Facultat de Farmàcia i Ciències de l’Alimentació, Campus de l’Alimentació de Torribera, Universitat de Barcelona (UB), Santa Coloma de Gramenet, Spain; ^2^Institut de Recerca en Nutrició i Seguretat Alimentària (INSA⋅UB), Universitat de Barcelona (UB), Santa Coloma de Gramenet, Spain; ^3^Xarxa d’Innovació Alimentària (XIA), Barcelona, Spain; ^4^Departament de Biologia, Sanitat i Mediambient, Secció de Microbiologia, Facultat de Farmàcia i Ciències de l’Alimentació, Universitat de Barcelona (UB), Barcelona, Spain

**Keywords:** histamine intolerance, intestinal microbiota, gut dysbiosis, low-histamine diet, DAO supplementation, histamine-secreting bacteria

## Abstract

Restrictive diets for the treatment of different gastrointestinal disorders are reported to change the composition of intestinal microbiota. Recently, it has been proposed that individuals with histamine intolerance suffer from intestinal dysbiosis, having an overabundance of histamine-secreting bacteria, but how it is still unknown this state is affected by the usual dietary treatment of histamine intolerance [i.e., low-histamine diet and the supplementation with diamine oxidase (DAO) enzyme]. Thus, a preliminary study was carried out aiming to evaluate the potential changes on the composition of the intestinal microbiota in a group of five women diagnosed with histamine intolerance undergoing 9 months of the dietary treatment of histamine intolerance. After sequencing bacterial 16S rRNA genes (V3-V4 region) and analyzing the data using the EzBioCloud Database, we observed a reduction in certain histamine-secreting bacteria, including the genera *Proteus* and *Raoultella* and the specie *Proteus mirabilis*. Moreover, it was also observed an increase in *Roseburia* spp., a bacterial group frequently related to gut health. These changes could help to explain the clinical improvement experienced by histamine intolerant women underwent a dietary treatment.

## Introduction

Dietary habits are among the external factors that exert the most influence on the composition of the intestinal microbiota (1–3). To date, several studies have evaluated the impact of different macro and micronutrients on intestinal microbial patterns (4–6). It has been demonstrated that a high consumption of animal proteins, saturated fat, sugar, and salt could weaken the intestinal barrier due to a higher growth of certain pathogenic bacteria (1). On the contrary, a diet rich in complex polysaccharides and plant protein could be related to an increase of certain health-related bacteria capable of stimulating the production of short-chain fatty acids (SCFA) in the intestines (1).

Several disorders affecting the gastrointestinal tract, such as food intolerances, irritable bowel syndrome (IBS) or celiac disease, can be managed by following highly restrictive diets (7–11). However, over time, these diets can have a negative impact on the composition of the intestinal microbiota, although this is a more complex relationship (11–15). In a recent study by Lenhart et al. IBS patients following low-FODMAP (Fermentable Oligosaccharides, Disaccharides, Monosaccharides, and Polyols), gluten-free or dairy-free diets showed significant differences in bacterial beta diversity and a reduction in the abundance of *Bifidobacterium*, *Lactobacillus*, and *Prevotella* genera (13). Similarly, De Palma et al. reported a reduction in the genera *Bifidobacterium* and *Lactobacillus* in healthy individuals following a gluten-free diet (14). These unwanted effects could be explained by the exclusion of FODMAPs and lactose, described as dietary carbohydrates with prebiotic actions, and gluten-containing foods such as wheat or barley, which are a source of prebiotic fructans (15–17).

Histamine intolerance is a food-related disorder caused by impaired histamine degradation at the intestinal level due to a deficiency in the enzyme diamine oxidase (DAO) (10). Affected individuals suffer a wide range of gastrointestinal and extraintestinal symptoms, such as bloating, diarrhea, abdominal pain, postprandial fullness, constipation, flatulences, headache, tachycardia, hypotonia, pruritus, eczema, urticaria, rhinitis, and nasal congestion. These manifestations usually appear after the consumption of foods containing histamine and/or other biogenic amines (18). In the last decade, new evidence regarding the aetiopathogenesis of histamine intolerance has been published (19–23). Several single nucleotide polymorphisms encoding a DAO enzyme with reduced histamine degradation capacity have been described as potential genetic causes of histamine intolerance (19, 23). Moreover, impaired DAO activity can also be temporary and reversible, being secondary to certain gastrointestinal disorders or as a side effect of some widely used pharmacological drugs (20). More recently, two studies have suggested that dysbiosis of the intestinal microbiota may play a role in this condition (24, 25). In 2018, Schink et al. demonstrated that patients with symptoms of histamine intolerance have an imbalance of the gut microbiota and an impaired intestinal barrier, which could lead to a deficiency in DAO catabolic activity (24). According to these authors, intestinal dysbiosis may contribute to mucosal inflammation and, in turn, favor the development of a leaky gut with the subsequent potential reduction of DAO enzymatic activity. Intestinal inflammation affecting mucosal integrity have been identified as a cause of low DAO activity by other previous works (26–28). In addition, it may be hypothesized that intestinal dysbiosis could be related to histamine intolerance by a possible higher presence of histaminogenic bacteria that could favor the accumulation of histamine at intestinal level, as well as, by a lower presence of bacteria with histaminolytic activity. In fact, the study performed by Mou et al. identified a total of 117 species from the human gut microbiome with the ability to form histamine (29). Moreover, Sánchez-Pérez et al. recently reported a higher proportion of histamine-secreting bacteria (e.g., *Staphylococcus*, *Proteus*, *Clostridium perfringens*, and *Enterococcus faecalis*) in patients with histamine intolerance in comparison with a healthy control group. These authors also reported alterations in gut bacterial diversity in histamine intolerant individuals (25). It would be important to consider that an intestinal dysbiosis could not be, by itself, the only cause of histamine intolerance but would probably aggravate the symptoms derived from other primary causes of a DAO deficiency (genetic or pathological). In fact, a dysbiosis could help explain the varying severity of symptoms frequently reported in individuals with histamine intolerance (30).

The usual dietary management of histamine intolerance is the follow-up of a low-histamine diet. These diets are based on the exclusion of histamine-containing foods (10, 31). As this amine in foods is mainly formed by the bacterial decarboxylation of the precursor aminoacid histidine, the foods susceptible to contain high histamine levels are those fermented or microbiologically altered (by the action of the fermentative bacteria or spoilage bacteria, respectively) (32, 33). Thus, dry-fermented sausages, cured cheese, and other fermented products, together with preserved and semi-preserved fish derivatives, can easily accumulate high histamine levels. Moreover, certain other foods, such as citrus fruits, strawberry, banana and nuts, that do not contain histamine but may contain other biogenic amines (e.g., putrescine and/or cadaverine) are also frequently excluded within low-histamine diets (31). The presence of these other amines can exert an inhibitory effect on the degradation of histamine by DAO enzyme due to the competition for this degradation system (18). Finally, the dietary treatment of histamine intolerance also considers the supplementation with exogenous DAO enzyme to enhance the intestinal degradation of histamine (10, 31).

There is still a lack of information about what happens to the gut microbiota when histamine intolerant patients are treated with a restrictive low-histamine diet and DAO supplementation. Therefore, a first preliminary study was carried out with the aim to evaluate potential changes on the composition of the intestinal microbiota in a group of five women undergoing the dietary treatment of histamine intolerance.

## Materials and methods

### Study design

A previous study was conducted in histamine intolerant patients and healthy individuals to characterize and compare the intestinal microbiota composition (25). The current pilot study was carried out with a subgroup of five women from the histamine intolerant group who presented at least three symptoms associated with histamine intolerance. The inclusion criteria were as follows: age between 18 and 65 years; diagnosis of histamine intolerance based on two or more symptoms and negative results for food allergen-specific IgE. The exclusion criteria were pregnancy, lactation, having started a low-histamine diet, and having taken antibiotics and/or probiotics the month before the study. Considering that the female sex seems prevalently affected by histamine intolerance (34), five women were included in the present pilot study. These women were patients from a nutrition and dietetic center specialized in the dietary management of histamine intolerance (DAO Deficiency Clinical Institute, Barcelona, Spain), where the follow-up of the dietary treatment, as well as the evolution of the symptomatology, were monitored. Table 1 displays the baseline characteristics of each patient (age, serum DAO activity, and symptoms). All women showed deficiency in serum DAO activity. The reported clinical manifestations were mainly gastrointestinal (i.e., bloating, heart burn, flatulence, diarrhea, and abdominal pain), followed by headache and dermatological complaints, such as pruritus and eczema. One patient also reported insomnia, muscular pain, and articular pain.

**TABLE 1:** Baseline characteristics of each patient with histamine intolerance.

Patient	Age (years)	DAO activity (U/mL)	Clinical symptoms
1	65	9.55	Headache, abdominal bloating, and heart burn.
2	31	6.50	Diarrhea, flatulences, and abdominal bloating.
3	44	9.80	Headache, diarrhea, abdominal bloating, articular pain, muscular pain, and insomnia
4	27	7.70	Diarrhea, abdominal pain, flatulences, and abdominal bloating.
5	56	9.15	Headache, eczema, and pruritus.

**FIGURE 1:**
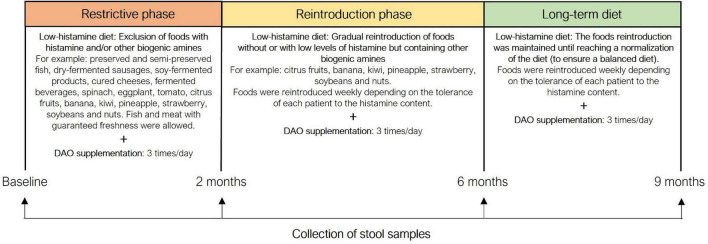
The different phases of the dietary treatment and stool sample collection during the 9-month study.

The current study aimed to evaluate the microbiota composition of these patients along 9-month of this dietary treatment (low-histamine diet and DAO supplementation). Figure 1 summarizes the study protocol, in which a total of four stool samples of each patient were collected: at baseline (before starting the dietary treatment) and at 2, 6, and 9 months of the study. Moreover, Figure 1 also details the phases of the dietary treatment prescribed by the nutrition and dietetic center specialized in the dietary management of histamine inolerance. The first phase was the shortest but also the most restrictive, involving the exclusion of all foods with histamine, and/or other biogenic amines. In the second phase, some excluded foods, mainly those without or with low histamine levels but containing other biogenic amines, were gradually reintroduced. Finally, in the last phase, the rest of excluded foods were progressively reintegrated as much as possible according to interindividual tolerance to histamine content in order to achieve a properly long-term balanced diet. During all phases, DAO supplements formulated with porcine kidney protein extract were administered 20 min prior to each main meal (Figure 1).

Plasma DAO activity was also analyzed using a Radio Extraction Assay (REA) according to the manufacturer’s instructions (Sciotec Diagnostic Technologies, Tulln, Austria). Values below 10 U/mL (Histamine Degrading Units) are considered as DAO deficient.

All participants were given detailed information about the aim and procedure of the study and gave their written informed consent prior to study inclusion. The study was approved by the Bioethics Committee of the University of Barcelona (IRB00003099).

### Determinations in stool samples

The composition of the gut microbiota was determined by isolation of the bacterial DNA from stool samples (QIAamp Power Fecal Pro DNA kit, QIAGEN, Germantown, MD, USA) and subsequent sequencing of the V3-V4 region of 16S rRNA (Illumina MiSeq platform) at the Genomic and Bioinformatic Service of the Autonomous University of Barcelona. Bioinformatics analysis of the microbiota composition was performed with the EzBiocloud Database (ChunLab, Inc., Seoul, Korea). For the 16S rRNA amplicons, baseline sequence data were previously deposited in the NCBI database by the Bioproject PRJNA811749 (25). The sequence data related to months 2, 6, and 9 of the dietary treatment were deposited in the NCBI database by the Bioproject PRJNA842201.

### Statistical analysis

Differences in the microbiota composition during the 9-month study were analyzed by the Kruskal–Wallis test for non-parametric data. Alpha diversity was measured using the Shannon index and Simpson’s index, and beta diversity was calculated by Bray–Curtis dissimilarity analysis and visualized using principal coordinates analysis (PCoA). *p*-values of *p* < 0.05 were considered statistically significant.

## Results and discussion

The follow-up of the improvement of the symptoms derived from the dietary treatment of each patient was carried out by the nutritionist of the dietetic center specialized in the dietary management of histamine intolerance. According to this information, all patients experienced a general improvement in the baseline symptoms. Gastrointestinal manifestations (i.e., bloating, diarrhea, flatulences, abdominal pain, and heart burn) were those that mainly disappeared during the dietary treatment most of them already from the second month. Headache improved in two out of the three patients that reported this symptom. Despite these improvements, any patient achieved a total remission of clinical manifestations.

Regarding the composition of the intestinal microbiota, a gut dysbiosis was evidenced in all the recruited women when compared with the results obtained for a group of healthy individuals used as a control group in the previous study performed by Sánchez-Pérez et al. (25). The observed statistically differences in the relative abundance of bacterial families, genera, and species are shown in Supplementary Table 1. In comparison with the control group, histamine intolerant patients showed a significantly higher relative abundance of some bacteria related with histamine-forming capacity (*Morganellaceae*, *Pseudomonadaceae*, *Staphylococcus*, *Proteus*, *Proteus mirabillis*, and *Clostridium perfringens*), as well as a reduction in the relative abundance of the genus *Faecalibacterium*, associated to a healthy gut (Supplementary Table 1).

**FIGURE 2:**
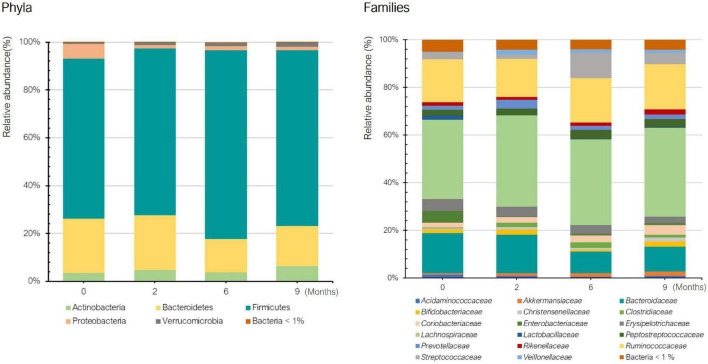
Relative abundance (%) of bacteria at phylum and family levels in histamine intolerant patients during the 9-month dietary management.

Figure 2 shows the mean relative abundance of bacteria at phylum and family levels in the histamine intolerant patients at baseline and during the dietary treatment (low-histamine diet and DAO supplementation). A similar phylum pattern was observed for all patients at all sampling points, Firmicutes and Bacteroides having the highest relative abundance (Figure 2). Overall, no statistically significant differences were found in phyla during the dietary management (*p* > 0.05). The high relative abundance of the phylum Proteobacteria observed at baseline is accounted for by the abnormal abundance of these bacteria in one specific patient (24%) in comparison with the other patients (1–2.5%). It is worth highlighting that this patient (patient 3) also had the highest number of symptoms (Table 1). An overgrowth of Proteobacteria has been observed in patients with different intestinal disorders, such as Crohn’s disease, ulcerative colitis, IBS and colorectal cancer, and has been postulated as a hallmark of dysbiosis (35–39). A marked reduction (90%) in the relative abundance of Proteobacteria was observed in this patient after 2 months of dietary treatment, when the values were similar to those of the other histamine intolerant patients. Changes in the abundance of Proteobacteria have been previously related to dietary habits (40). Levine et al. also reported a reduced relative abundance of this phylum in pediatric patients with Crohn’s disease after they followed a restrictive diet (i.e., excluding foods containing wheat, dairy, animal fats, and additives) and received enteral nutrition (41).

**FIGURE 3:**
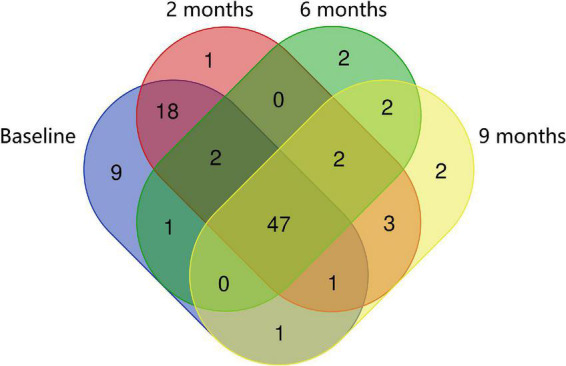
Venn diagrams of specific and shared Operational Taxonomic Units (OTUs) detected at the family level at baseline, 2, 6, and 9 months of the study.

**FIGURE 4:**
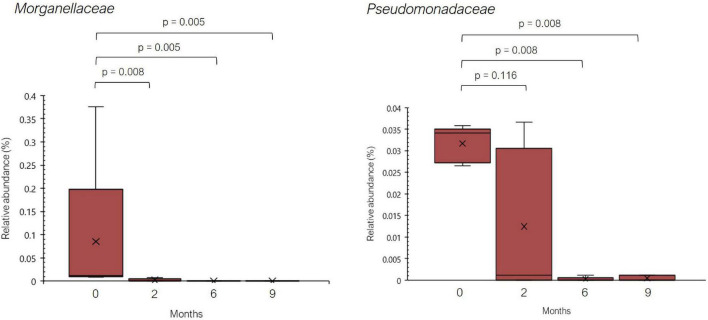
Relative abundance (%) of *Morganellaceae* and *Pseudomonadaceae* observed in histamine intolerant patients during the dietary treatment. Mean values are indicated by an ×. *p* < 0.05 indicates statistically significant differences.

Regarding bacterial families, the distribution pattern in the histamine intolerant patients showed few variations during the dietary treatment (Figure 2). Nevertheless, statistically significant changes were observed in the relative abundance of 21 bacterial families, all of them with relative abundances below 1%. These bacterial families are listed in Supplementary Table 2. Moreover, a reduction on the total number of families was found, decreasing from a total of 79 bacterial families at baseline to 58 at 9 months, some of them related to non-desirable effects (Figure 3 and Supplementary Table 2). For example, the family *Morganellaceae* was considerably reduced in all patients at the second month of the study and absent at 6 months (Figure 4). *Morganellaceae* includes bacterial species with a high histamine-formation capacity, such as *Morganella morganii* (29, 42–44). *Pseudomonadaceae* also showed a marked reduction in the relative abundance, especially after 6 months of dietary management (Figure 4). High levels of pseudomonas bacteria have been associated with cases of inflammatory bowel disease, also being a bacterial family with several strains related with histamine production (45–50).

**FIGURE 5:**
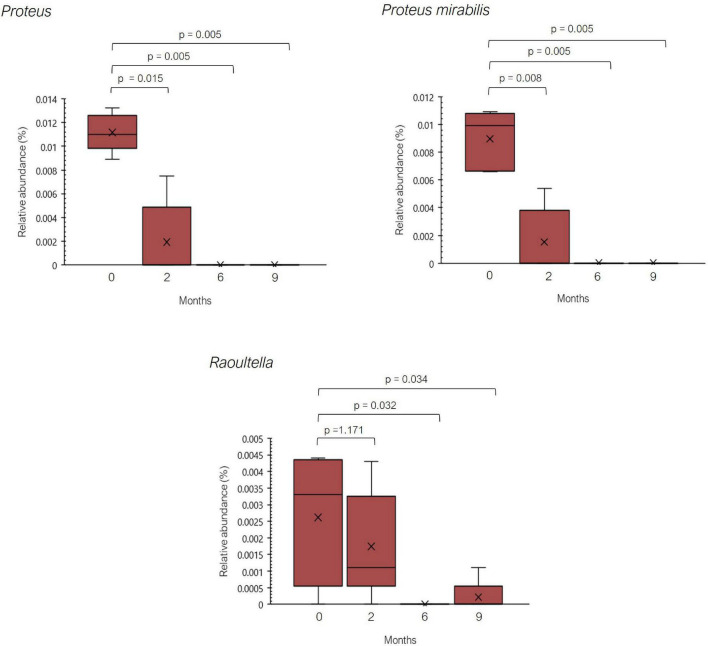
Relative abundance (%) of *Proteus*, *Proteus mirabilis*, and *Raoultella* observed in histamine intolerant patients during the dietary management. Mean values are represented with an ×. *p* < 0.05 indicates statically significant differences.

In the previous study performed by Sánchez-Pérez et al. a greater relative abundance of several histaminogenic bacteria, mainly belonging to the family *Enterobacteriaceae* and to the genera *Staphylococcus* and *Proteus*, was reported in histamine intolerant individuals in contrast with the healthy control group (25). Thus, an excessive accumulation of bacterial-derived histamine at the intestinal level could account for the onset of symptoms associated with histamine intolerance. In the present study, statistically significant changes were observed in the relative abundance of 44 genera and 64 species during the 9-month dietary treatment (Supplementary Tables 3, 4). Among them, it is worth highlighting the significant reduction observed in the relative abundance of bacteria with recognized histamine-secreting ability (29, 42–44, 51, 52). For example, the relative abundance of the genus *Proteus* and the species *Proteus mirabilis* was dramatically reduced at the second month of the study, being absent in all patients after the sixth month (Figure 5). For the genus *Raoultella*, this reduction was statistically significant at 6 and 9 months. In fact, Mou et al. identified some species of the genus *Raoultella* with a putative histamine-secreting capacity within the human gut microbiome (29).

**FIGURE 6:**
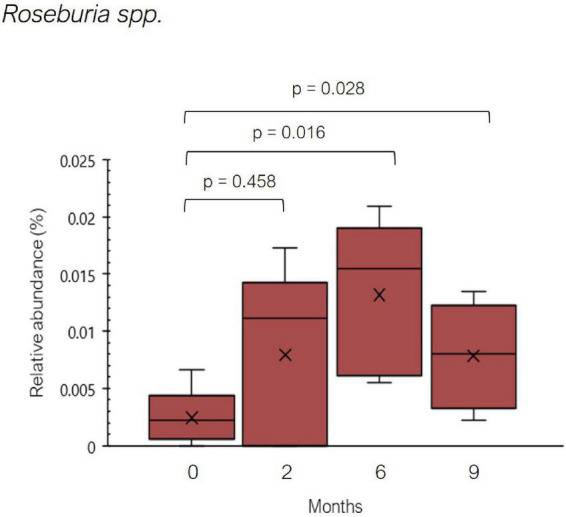
Relative abundance (%) of *Roseburia* spp. found in histamine intolerant patients during the dietary treatment. Mean values are represented with an ×. *p* < 0.05 indicates statically significant differences.

On the other hand, a significant increase in the relative abundance of *Roseburia* spp. was observed throughout the dietary treatment (Figure 6). In addition to the reduction of histamine-secreting bacteria, it can be speculated that the decline in number of symptoms reported by the five histamine intolerant women could be partially explained by the increase in *Roseburia* spp. Beneficial properties have been attributed to *Roseburia* species due to its capacity to produce SCFA, which are involved in the maintenance of intestinal homeostasis and the inhibition of a proinflammatory status (53–55). A high abundance of SCFA-producers, among them *Roseburia*, has been found in individuals on diets in which animal-derived products are replaced with plant-based foods (56–58). Low-histamine diets tend to be richer in plant-derived products, as they aim to reduce the consumption of meat and fish derivatives and cured and raw milk cheeses.

Finally, regarding bacterial diversity, no changes were observed for any patient during the dietary treatment in terms of alpha diversity (evaluated by the Shannon and Simpson indices) (*p* > 0.05). In the case of beta diversity (assessed by Bray-Curtis dissimilarity), no significant changes were observed in interindividual differences in the distribution patterns of genera and species along the dietary treatment. In the previous work performed by Sánchez-Pérez et al. statistical differences were observed in the beta diversity between intolerant individuals and the healthy group, this latter showing more homogenous microbial patterns among them (25). In the current study, after 9 months on a low-histamine diet associated with DAO supplementation, 3 out of 5 histamine intolerant patients showed a pattern of microbial species that clustered closely with that previously reported for healthy individuals (25).

These preliminary results prompt further studies to assess the positive effect of the dietary treatment on the gut microbiota composition in histamine intolerant patients, with a larger sample set and involving healthy individuals undergoing the same dietary treatment. The lack of male representation, of data on daily food consumption, as well as regarding the changes in symptom intensity, should also be addressed in future works.

Despite the limitations of the study, preliminary results suggest that the usual dietary treatment of histamine intolerance may influence the composition of intestinal microbiota in histamine intolerant women. According to our results, a reduction in the relative abundance of histamine-secreting bacteria such as *Pseudomonadaceae*, *Proteus*, *Proteus mirabilis*, and *Raoultella* was observed during the dietary treatment. These results would support the hypothesis that a reduction in histamine-secreting bacteria could diminish the accumulation and absorption of histamine at intestinal level and, subsequently, avoid the onset of symptoms. Likewise, it was observed an increase in *Roseburia* spp., bacteria related to an improvement of the inflammation of intestinal mucosa. According to our knowledge, this is the first time that a follow-up study has been performed to assess the influence of the dietary treatment of histamine intolerance on intestinal microbiota composition. The majority of the available studies that evaluate the efficacy of a low-histamine diet on the symptoms of this intolerance considered intervention periods of up to 3 months while in this study, despite the low number of patients, 9-months of treatment were considered. Further studies with a higher number of patients are needed to better understand the relationship between the dietary treatment of histamine intolerance and changes in intestinal microbiota composition. In addition, considering the existence of certain technological bacterial strains with DAO activity it would be interesting to explore the use of DAO-positive probiotic bacteria for the preventive treatment of histamine intolerance (59).

## Data availability statement

The datasets presented in this study can be found in online repositories. The names of the repository/repositories and accession number(s) can be found below: https://www.ncbi.nlm.nih.gov/, Bioproject PRJNA842201.

## Ethics statement

The studies involving human participants were reviewed and approved by the Bioethics Committee of the University of Barcelona (IRB00003099). The patients/participants provided their written informed consent to participate in this study.

## Author contributions

MV-N, ML-M, and MV-C: conceptualization. SS-P, OC-B, and AD: investigation. SS-P, OC-B, MB, and ML-M: data curation and writing—original draft preparation. SS-P, OC-B, AD, MV-N, MB, ML-M, and MV-C: writing—review and editing. ML-M and MV-C: supervision. All authors have read and agreed to the published version of the manuscript.

## References

[1] Rinninella E Cintoni M Raoul P Lopetuso LR Scaldaferri F Pulcini G Food components and dietary habits: keys for a healthy gut microbiota composition. *Nutrients.* (2019) 11:2393. 10.3390/nu11102393 31591348 PMC6835969

[2] Power SE O’Toole PW Stanton C Ross RP Fitzgerald GF. Intestinal microbiota, diet and health. *Br J Nutr.* (2014) 111:387–402. 10.1017/S0007114513002560 23931069

[3] Salonen A de Vos WM. Impact of diet on human intestinal microbiota and health. *Ann Rev Food Sci Technol.* (2014) 5:239–62. 10.1146/annurev-food-030212-182554 24387608

[4] Singh A Zapata RC Pezeshki A Reidelberger RD Chelikani PK. Inulin fiber dose-dependently modulates energy balance, glucose tolerance, gut microbiota, hormones and diet preference in high-fat-fed male rats. *J Nutr Biochem.* (2018) 59:142–52. 10.1016/j.jnutbio.2018.05.017 30005919

[5] Wolters M Ahrens J Romaní-Pérez M Watkins C Sanz Y Benítez-Páez A Dietary fat, the gut microbiota, and metabolic health – a systematic review conducted within the my new gut project. *Clin Nutr.* (2019) 38:2504–20. 10.1016/j.clnu.2018.12.024 30655101

[6] Rinninella E Mele MC Merendino N Cintoni M Anselmi G Caporossi A The role of diet, micronutrients and the gut microbiota in age-related macular degeneration: new perspectives from the gut–retina axis. *Nutrients.* (2018) 10:1677. 10.3390/nu10111599 30400586 PMC6267253

[7] Fassio F Facioni MS Guagnini F. Lactose maldigestion, malabsorption, and intolerance: a comprehensive review with a focus on current management and future perspectives. *Nutrients.* (2018) 10:1599. 10.3390/nu10111599 30388735 PMC6265758

[8] Bardisi BM Halawani AKH Halawani HKH Alharbi AH Turkostany NS Alrehaili TS Efficiency of diet change in irritable bowel syndrome. *J Family Med Prim Care.* (2018) 7:946–51. 10.4103/jfmpc.jfmpc_173_18 30598938 PMC6259499

[9] Sansotta N Amirikian K Guandalini S Jericho H. Celiac disease symptom resolution: effectiveness of the gluten-free diet. *J Pediatr Gastroenterol Nutr.* (2018) 66:48–52. 10.1097/MPG.0000000000001634 28514243

[10] Comas-Basté O Sánchez-Pérez S Veciana-Nogués MT Latorre-Moratalla M Vidal-Carou MC. Histamine intolerance: the current state of the art. *Biomolecules.* (2020) 10:1–26. 10.3390/biom10081181 32824107 PMC7463562

[11] Staudacher HM Whelan K Irving PM Lomer MCE. Comparison of symptom response following advice for a diet low in fermentable carbohydrates (FODMAPs) versus standard dietary advice in patients with irritable bowel syndrome. *J Hum Nutr Diet.* (2011) 24:487–95. 10.1111/j.1365-277X.2011.01162.x 21615553

[12] Starz E Wzorek K Folwarski M Kaźmierczak-Siedlecka K Stachowska L Przewłócka K The modification of the gut microbiota via selected specific diets in patients with crohn’s disease. *Nutrients.* (2021) 13:2125. 10.3390/nu13072125 34206152 PMC8308385

[13] Lenhart A Dong T Joshi S Jaffe N Choo C Liu C Effect of exclusion diets on symptom severity and the gut microbiota in patients with irritable bowel syndrome. *Clin Gastroenterol Hepatol.* (2022) 20:465–83. 10.1016/j.cgh.2021.05.027 34022450 PMC9054035

[14] De Palma G Nadal I Collado MC Sanz Y. Effects of a gluten-free diet on gut microbiota and immune function in healthy adult human subjects. *Br J Nutr.* (2009) 102:1154–60. 10.1017/S0007114509371767 19445821

[15] Gibson PR Halmos EP Muir JG. Review article: fodmaps, prebiotics and gut health-the fodmap hypothesis revisited. *Aliment Pharmacol Ther.* (2020) 52:233–46. 10.1111/apt.15818 32562590

[16] Salli K Anglenius H Hirvonen J Hibberd AA Ahonen I Saarinen MT The effect of 2’-fucosyllactose on simulated infant gut microbiome and metabolites; a pilot study in comparison to gos and lactose. *Sci Rep.* (2019) 9:13232. 10.1038/s41598-019-49497-z 31520068 PMC6744565

[17] Jackson FW. Effects of a gluten-free diet on gut microbiota and immune function in healthy adult human subjects - comment by jackson. *Br J Nutr.* (2010) 104:773. 10.1017/S0007114510001960 20465858

[18] Sánchez-Pérez S Comas-Basté O Costa-Catala J Iduriaga-Platero I Veciana-Nogués MT Vidal-Carou MC The rate of histamine degradation by diamine oxidase is compromised by other biogenic amines. *Front Nutr.* (2022) 9:897028. 10.3389/fnut.2022.897028 PMC9175030 35694170

[19] Maintz L Yu CF Rodríguez E Baurecht H Bieber T Illig T Association of single nucleotide polymorphisms in the diamine oxidase gene with diamine oxidase serum activities. *Allergy.* (2011) 66:893–902. 10.1111/j.1398-9995.2011.02548.x 21488903

[20] Leitner R Zoernpfenning E Missbichler A. Evaluation of the inhibitory effect of various drugs / active ingredients on the activity of human diamine oxidase in vitro. *Clin Transl Allergy.* (2014) 4:23. 10.1186/2045-7022-4-s3-p23

[21] Enko D Kriegshäuser G Halwachs-Baumann G Mangge H Schnedl WJ. Serum diamine oxidase activity is associated with lactose malabsorption phenotypic variation. *Clin Biochem.* (2017) 50:50–3. 10.1016/j.clinbiochem.2016.08.019 27593109

[22] Schnedl WJ Enko D. Considering histamine in functional gastrointestinal disorders. *Crit Rev Food Sci Nutr.* (2020) 61:2960–7. 10.1080/10408398.2020.1791049 32643952

[23] García-Martín E García-Menaya J Sánchez B Martínez C Rosendo R Agúndez JAG. Polymorphisms of histamine-metabolizing enzymes and clinical manifestations of asthma and allergic rhinitis. *Clin Exp Allergy.* (2007) 37:1175–82. 10.1111/j.1365-2222.2007.02769.x 17651147

[24] Schink M Konturek PC Tietz E Dieterich W Pinzer TC Wirtz S Microbial patterns in patients with histamine intolerance. *J Physiol Pharmacol.* (2018) 69:579–93. 10.26402/jpp.2018.4.09 30552302

[25] Sánchez-Pérez S Comas-Basté O Duelo A Veciana-Nogués MT Berlanga M Latorre-Moratalla ML Intestinal dysbiosis in patients with histamine intolerance. *Nutrients.* (2022) 14:1774. 10.3390/nu14091774 35565742 PMC9102523

[26] Fukudome I Kobayashi M Dabanaka K Maeda H Okamoto K Okabayashi T Diamine oxidase as a marker of intestinal mucosal injury and the effect of soluble dietary fiber on gastrointestinal tract toxicity after intravenous 5-fluorouracil treatment in rats. *Med Mol Morphol.* (2014) 47:100–7. 10.1007/s00795-013-0055-7 24005798

[27] Mondovi B Fogel WA Federico R Calinescu C Mateescu MA Rosa AC Effects of amine oxidases in allergic and histamine-mediated conditions. *Recent Pat. Inflamm Allergy Drug Discov.* (2013) 7:20–34. 22946464

[28] Enko D Meinitzer A Mangge H Kriegshaüser G Halwachs-Baumann G Reininghaus EZ Concomitant prevalence of low serum diamine oxidase activity and carbohydrate malabsorption. *Can J Gastroentero Hepatol.* (2016) 2016:1–4. 10.1155/2016/4893501 28042564 PMC5155086

[29] Mou Z Yang Y Hall AB Jiang X. The taxonomic distribution of histamine-secreting bacteria in the human gut microbiome. *BMC Genomics.* (2021) 22:695. 10.1186/s12864-021-08004-3 34563136 PMC8465708

[30] Schnedl WJ Lackner S Enko D Schenk M Holasek SJ Mangge H. Evaluation of symptoms and symptom combinations in histamine intolerance. *Intest Res.* (2019) 17:427–33. 10.5217/ir.2018.00152 30836736 PMC6667364

[31] Sánchez-Pérez S Comas-Basté O Veciana-Nogués MT Latorre-Moratalla ML Vidal-Carou MC. Low-histamine diets: is the exclusion of foods justified by their histamine content? *Nutrients.* (2021) 13:1395. 10.3390/nu13051395 33919293 PMC8143338

[32] European Food Safety Authority [EFSA]. Panel on biological hazard (BIOHAZ). Scientific opinion on risk based control of biogenic amine formation in fermented foods. *EFSA J.* (2011) 9:1–93. 10.2903/j.efsa.2011.2393

[33] Comas-Basté O Latorre-Moratalla ML Sánchez-Pérez S Veciana-Nogués MT Vidal-Carou MC. Histamine and other biogenic amines in food. In: Proestos C editor. *From scombroid poisoning to histamine intolerance.* Biogenic Amines, London: IntechOpen (2019).

[34] Hrubisko M Dani D Huorka M Wawruch M. Histamine intolerance – the more we know the less we know. *Nutrients.* (2021) 13:2228. 10.3390/nu13072228 34209583 PMC8308327

[35] Gophna U Sommerfeld K Gophna S Doolittle WF Veldhuyzen van Zanten SJ. Differences between tissue-associated intestinal microfloras of patients with crohn’s disease and ulcerative colitis. *J Clin Microbiol.* (2006) 44:4136–41. 10.1128/JCM.01004-06 16988016 PMC1698347

[36] Wang T Cai G Qiu Y Fei N Zhang M Pang X Structural segregation of gut microbiota between colorectal cancer patients and healthy volunteers. *ISME J.* (2012) 6:320–9. 10.1038/ismej.2011.109 21850056 PMC3260502

[37] Krogius-Kurikka L Lyra A Malinen E Aarnikunnas J Tuimala J Paulin L Microbial community analysis reveals high level phylogenetic alterations in the overall gastrointestinal microbiota of diarrhoea-predominant irritable bowel syndrome sufferers. *BMC Gastroenterol.* (2009) 9:1–1. 10.1186/1471-230X-9-95 20015409 PMC2807867

[38] Morgan XC Tickle TL Sokol H Gevers D Devaney KL Ward DV Dysfunction of the intestinal microbiome in inflammatory bowel disease and treatment. *Genome Biol.* (2012) 13:79. 10.1186/gb-2012-13-9-r79 23013615 PMC3506950

[39] Litvak Y Byndloss MX Tsolis RM Bäumler AJ. Dysbiotic *Proteobacteria* expansion: a microbial signature of epithelial dysfunction. *Curr Opin Microbiol.* (2017) 39:1–6. 10.1016/j.mib.2017.07.003 28783509

[40] De Filippo C Cavalieri D Di Paola M Ramazzotti M Poullet JB Massart S. Impact of diet in shaping gut microbiota revealed by a comparative study in children from Europe and rural Africa. *Proc Natl Acad Sci USA.* (2010) 107:14691–6. 10.1073/pnas.1005963107 20679230 PMC2930426

[41] Levine A Wine E Assa A Sigall Boneh R Shaoul R Kori M Crohn’s disease exclusion diet plus partial enteral nutrition induces sustained remission in a randomized controlled trial. *Gastroenterology.* (2019) 157:440–50. 10.1053/j.gastro.2019.04.021 31170412

[42] Bjornsdottir-Butler K Bolton GE Mcclellan-Green PD Jaykus LA Green DP. Detection of gram-negative histamine-producing bacteria in fish: a comparative study. *J Food Protec.* (2009) 72:1987–91. 10.4315/0362-028x-72.9.1987 19777904

[43] Bjornsdottir-Butler K Jones JL Benner R Burkhardt W. Development of a real-time PCR assay with an internal amplification control for detection of gram-negative histamine-producing bacteria in fish. *Food Microbiol.* (2011) 28:356–63. 10.1016/j.fm.2010.06.013 21356438

[44] Ladero V Calles-Enríquez M Fernández M Alvarez MA. Toxicological effects of dietary biogenic amines. *Curr Nutr Food Sci.* (2010) 6:145–56. 10.2174/157340110791233256 26958625

[45] Mukhopadhya I Hansen R El-Omar EM Hold GL. IBD-what role do *Proteobacteria* play? *Nat Rev Gastroenterol Hepatol.* (2012) 9:219–30. 10.1038/nrgastro.2012.14 22349170

[46] Wagner J Short K Catto-Smith AG Cameron DJS Bishop RF Kirkwood CD. Identification and characterization of *Pseudomonas* 16s ribosomal dna from ileal biopsies of children with Crohn’s disease. *PLoS One.* (2008) 3:3578. 10.1371/journal.pone.0003578 18974839 PMC2572839

[47] Parent K Mitchell P. *Pseudomonas*-like group Va bacteria in Crohn’s disease. *Gastroenterology.* (1978) 75:765. 710845

[48] Parent K Mitchell P. Cell wall-defective variants of *Pseudomonas*-like (group Va) bacteria in Crohn’s disease. *Gastroenterology.* (1978) 75:368–72. 10.1016/0016-5085(78)90834-x 680490

[49] Wei B Huang T Dalwadi H Sutton CL Bruckner D Braun J. *Pseudomonas fluorescens* encodes the crohn’s disease-associated i2 sequence and t-cell superantigen. *Infect Immun.* (2002) 70:6567–75. 10.1128/IAI.70.12.6567-6575.2002 12438326 PMC133002

[50] Lavizzari T Veciana-Nogués MT Weingart O Bover-Cid S Mariné-Font A Vidal-Carou MC. Occurrence of biogenic amines and polyamines in spinach and changes during storage under refrigeration. *J Agric Food Chem.* (2007) 55:9514–9. 10.1021/jf071307l 17935290

[51] Kanki M Yoda T Tsukamoto T Shibata T. *Klebsiella pneumoniae* produces no histamine: *Raoultella planticola* and *Raoultella omithinolytica* strains are histamine producers. *Appl Environ Microbiol.* (2002) 68:3462–6. 10.1128/AEM.68.7.3462-3466.2002 12089029 PMC126807

[52] Sêkowska A. Raoultella spp.—clinical significance, infections and susceptibility to antibiotics. *Folia Microbiol.* (2017) 62:221–7. 10.1007/s12223-016-0490-7 28063019

[53] Tamanai-Shacoori Z Smida I Bousarghin L Loreal O Meuric V Fong SB *Roseburia* spp.: a marker of health? *Future Microbiol.* (2017) 12:157–70. 10.2217/fmb-2016-0130 28139139

[54] Venegas DP de La Fuente MK Landskron G González MJ Quera R Dijkstra G. Short chain fatty acids (SCFAs) mediated gut epithelial and immune regulation and its relevance for inflammatory bowel diseases. *Front Immunol.* (2019) 10:277. 10.3389/fimmu.2019.00277 30915065 PMC6421268

[55] Fernández J Redondo-Blanco S Gutiérrez-del-Río I Miguélez EM Villar CJ Lombó F. Colon microbiota fermentation of dietary prebiotics towards short-chain fatty acids and their roles as anti-inflammatory and antitumour agents: a review. *J Funct Foods.* (2016) 25:511–22. 10.1016/j.jff.2016.06.032

[56] David LA Maurice CF Carmody RN Gootenberg DB Button JE Wolfe BE Diet rapidly and reproducibly alters the human gut microbiome. *Nature.* (2014) 505:559–63. 10.1038/nature12820 24336217 PMC3957428

[57] Tomova A Bukovsky I Rembert E Yonas W Alwarith J Barnard ND. The effects of vegetarian and vegan diets on gut microbiota. *Front Nutr.* (2019) 6:47. 10.3389/fnut.2019.00047 31058160 PMC6478664

[58] Dahl WJ Rivero-Mendoza D Lambert JM. Diet, nutrients and the microbiome. *Prog Mol Biol Transl Sci.* (2020) 171:237–63. 10.1016/bs.pmbts.2020.04.006 32475524

[59] Alvarez MA Moreno-Arribas MV. The problem of biogenic amines in fermented foods and the use of potential biogenic amine-degrading microorganisms as a solution. *Trends Food Sci Technol.* (2014) 39:146–55. 10.1016/j.tifs.2014.07.007

